# Correction: Assessing immunogenicity of CRISPR‑NCas9 engineered strain against porcine epidemic diarrhea virus

**DOI:** 10.1007/s00253-024-13192-5

**Published:** 2024-05-24

**Authors:** Fengsai Li, Haiyuan Zhao, Ling Sui, Fangjie Yin, Xinzi Liu, Guihai Guo, Jiaxuan Li, Yanping Jiang, Wen Cui, Zhifu Shan, Han Zhou, Li Wang, Xinyuan Qiao, Lijie Tang, Xiaona Wang, Yijing Li

**Affiliations:** 1 College of Veterinary Medicine, Northeast Agricultural University, Harbin, 150030 China; 2 Hebei Key Laboratory of Preventive Veterinary Medicine, College of Animal Science and Technology, Hebei Normal University of Science and Technology, Qinhuangdao, 066004 China; 3 Heilongjiang Key Laboratory for Animal Disease Control and Pharmaceutical Development, Harbin, 150030 China

**Correction** **:** **Applied Microbiology and Biotechnology**

10.1007/s00253-023-12989-0

Mistakes have been identified in the affiliation links of the original published version of this article.

The incorrect affiliation and affiliation links are shown below:

Fengsai Li^1^, Haiyuan Zhao^2^, Ling Sui^2^, Fangjie Yin^2^, Xinzi Liu^2^, Guihai Guo^2^, Jiaxuan Li^2,3^, Yanping Jiang^2,3^, Wen Cui^2,3^, Zhifu Shan^2,3^, Han Zhou^2,3^, Li Wang^2,3^, Xinyuan Qiao^2,3^, Lijie Tang^2,3^, Xiaona Wang^2,3^, Yijing Li^2,3^

^1^College of Veterinary Medicine, Northeast Agricultural University, Harbin 150030, China

^2^Hebei Key Laboratory of Preventive Veterinary Medicine, College of Animal Science and Technology, Hebei Normal University of Science and Technology, Qinhuangdao 066004, China

^3^Heilongjiang Key Laboratory for Animal Disease Control and Pharmaceutical Development, Harbin 150030, China

The correct affiliation links have been reflected in the author names group and the original article has been corrected.

